# Does polycystic ovary syndrome affect self-confidence and happiness?

**DOI:** 10.1590/1806-9282.20251064

**Published:** 2026-01-09

**Authors:** Nurseli Soylu Erener, İpek Turhan, Evrim Bayraktar

**Affiliations:** 1 Erciyes University, Faculty of Health Sciences, Department of Obstetrics and Gynecological Nursing – Kayseri, Turkey.; 2 Kayseri City Hospital – Kayseri, Turkey.

**Keywords:** Polycystic ovary syndrome, Quality of life, Happiness

## Abstract

**OBJECTIVE:**

The aim of this study was to examine the relationship between quality of life and self-confidence in women diagnosed with polycystic ovary syndrome, a chronic endocrine disorder that affects both physical and psychological well-being.

**METHODS:**

A cross-sectional study was conducted with women diagnosed with polycystic ovary syndrome. Data were collected using the Polycystic Ovary Syndrome Quality of Life-50 Scale (PCOSQ-50) and the Female Self-Confidence Scale. Descriptive statistics, correlation, and regression analyses were used to evaluate the association between quality of life and self-confidence levels.

**RESULTS:**

The findings indicated that sociodemographic and gynecologic characteristics did not significantly affect the quality of life or self-confidence levels of the participants. However, a significant positive correlation was observed between polycystic ovary syndrome-related quality of life and self-confidence (p>0.05). Women with lower quality of life scores reported significantly lower levels of self-confidence. Psychological distress, body image dissatisfaction, and infertility concerns were found to be key contributing factors to decreased self-confidence.

**CONCLUSION:**

Polycystic ovary syndrome negatively impacts women's psychological well-being and self-confidence, in addition to its physical symptoms. Integrating psychological support into polycystic ovary syndrome treatment may help to improve quality of life and promote emotional resilience. A multidisciplinary approach is essential for comprehensive care of women with polycystic ovary syndrome.

## INTRODUCTION

Polycystic ovary syndrome (PCOS) represents one of the most prevalent endocrine disorders affecting women of reproductive age. Under the Rotterdam criteria, a diagnosis is confirmed when at least two of the following clinical or diagnostic features are present: oligomenorrhea or amenorrhea, clinical and/or biochemical hyperandrogenism, and polycystic ovarian morphology identified through ultrasonography^1^. The condition is estimated to affect approximately 15–20% of women worldwide^1^.

Beyond its well-documented metabolic and physiological implications—such as insulin resistance, obesity, dyslipidemia, and an elevated risk of cardiovascular disease—PCOS is also closely linked to a range of psychological and emotional difficulties^2^. Physical manifestations, including hirsutism, acne, and androgenic alopecia, may compromise body image and diminish self-esteem in affected women^3^. Furthermore, obesity can impose challenges in social engagement and family dynamics, ultimately leading to decreased life satisfaction^4^.

Self-esteem is widely recognized as a core determinant of psychological well-being. In women with PCOS, diminished self-esteem may be accompanied by persistent feelings of unhappiness and worthlessness^5^. Against this backdrop, the present study seeks to explore the association between self-esteem and happiness levels among women diagnosed with PCOS.

## METHODS

This study is a descriptive study. Data were collected from all volunteer women who applied to the University Health Practice and Research Center Gynecology Outpatient Clinic and met the inclusion criteria. Since the population of the study was unknown, the study sample consisted of all women who came to the outpatient clinic between 06.02.2023 and 04.09.2023. The study was completed with a total of 203 women who met the inclusion criteria.

### Inclusion criteria

- 18 years of age or older
- Diagnosed with PCOS
- Literate
- Oriented to place, time, and situation
- Those who voluntarily agreed to participate in the study

### Exclusion criteria

- Women with psychiatric illness (bipolar disorder, depression, and obsessive compulsive disorder)

### Termination criteria

- Women who declined to participate in the research
- Women with missing data

### Data collection

In the study, after the necessary explanation about the research was made and the informed voluntary consent of the participants was obtained, the Patient Introduction Form, "PCOSQ-50 Scale," "Female Self-Confidence Scale," and "Oxford Happiness Scale."

### Patient Introduction Form

The study was designed to determine the sociodemographic (age, education level, employment status, occupation, income level, and duration of cohabitation with spouse/sexual partner), obstetric and gynecologic characteristics (when PCOS was diagnosed, number of pregnancy, delivery, curettage, and miscarriage) of women diagnosed with PCOS and consisted of 11 questions in total.

### Polycystic Ovary Syndrome Quality of Life-50 Scale

The Turkish validity and reliability study of the PCOSQ-50, originally developed by Nasiri-Amiri et al., was conducted by Koyutürk^5,6^. The scale demonstrated high internal consistency, with a Cronbach's alpha of 0.972; subdimensions ranged from 0.956 to 0.977. It comprises six subdimensions: psychosocial-emotional, fertility, sexual function, obesity and menstrual disorders, hair growth, and coping. The 50 items assess the frequency of experiences over the past four weeks, rated from 5 ("never") to 1 ("always"). Total scores range from 50 to 250. Unanswered items are excluded from the calculation^6^.

### Female Self-Confidence Scale

Developed by Yurtçiçek Ergüntop in 2019, this 38-item, five-point Likert-type scale measures women's self-confidence across five sub-dimensions^7^. The total Cronbach's alpha was reported as 0.97, with subdimensions ranging between 0.77 and 0.94. Higher scores indicate greater levels of self-confidence^7^.

### Oxford Happiness Questionnaire—Short Form

Originally created by Argyle et al. in 1989, the scale was revised into a short form by Hills and Argyle in 2002^8^. The Turkish adaptation was performed by Doğan et al^9^. This seven-item, five-point Likert-type scale yields scores between 7 and 35, with higher scores reflecting greater happiness^9^.

### Ethical aspects

Ethics Committee Permission from the University Social and Human Sciences Ethics Committee, Institutional Permission from the University Health Application and Research Center and academic board decision from the Faculty of Health Sciences were obtained for the research. The women who volunteered to participate in the study were given the necessary explanations about the research and signed an informed consent form. In the data collected online, an explanation section was included before the survey questions, and it was stated that the relevant option was marked to indicate that they agreed to participate in the study.

In this study, the Cronbach's alpha value of the PCOSQ-50 scale was found to be 0.949.

In this study, the Cronbach's alpha value of the Female Self-Confidence Scale was found to be 0.946.

### Data evaluation

While evaluating the data obtained from the study, SPSS (Statistical Package for the Social Sciences) for Windows 22.0 package program will be used for statistical analysis. Descriptive statistics number of units (n), percentage (%), and mean±standard deviation ( $\bar{\text{x}}\pm \text{ss}$ ) were used. The normal distribution of numerical variables was evaluated by Shapiro-Wilk normality test and Q–Q plot graphs. In two-group comparisons, independent-t test was used for variables with normal distribution and Mann-Whitney U test was used for variables without normal distribution. In comparisons of three or more groups, Kruskal-Wallis test was used for variables not showing normal distribution and ANOVA test was used for variables showing normal distribution. Cronbach's alpha internal consistency coefficients were calculated for the internal consistency of the scales. The relationships between the scales were evaluated by correlation analysis. A value of p<0.05 was considered statistically significant.

In this study, the Cronbach's alpha value of the subdimensions was found to be 0.900 for the psychosocial and emotional subdimension, 0.900 for the fertility subdimension, 0.900 for the sexual function subdimension, 0.900 for the obesity and menstrual disorders subdimension, and 0.900 for the hair growth and coping subdimension, respectively.

## RESULTS

It was found that the mean score of the PCOSQ-50 scale was 135.93±37.77 and the mean score of the Female Self-Confidence Scale was 137.17±24.20.

The characteristics of the women who participated in the study are given Table 1. The characteristics of the women and their PCOSQ-50 and Female Self-Confidence Scale scores are given in Table 2. It was found that 46.3% of the women were under 25 years of age, 45.8% had a PCOS diagnosis period of 1–4 years, and 85.2% were university/graduate graduates. It was determined that 83.7% of the women were employed, 70.0% perceived their income status as medium, 75.4% lived in the city center, and 54.7% were single. It was determined that 53.2% of the women who participated in the study had a sexual partner, 83.3% had no experience of pregnancy, 89.2% had no experience of birth/living child, 95.6% had no experience of abortion, and 91.6% had no experience of miscarriage. It was determined that the variables such as age, duration of PCOS diagnosis, employment status, birth/abortion/abortion experience, etc. did not statistically affect the PCOSQ-50 and Female Self-Confidence Scale scores of women (p>0.05).

**Table 1:** Characteristics of the women who participated in the study.

Features		N	%
Age			
	Under 25	94	46.3
	25–30 years	75	36.9
	Over 31 years	34	16.7
PCOS diagnosis period			
	1–4 years	93	45.8
	5–9 years	48	23.6
	10 years and over	62	30.5
Education status			
	High school and below	30	14.8
	University/master's degree	173	85.2
Employment status			
	No	33	16.3
	Yes	170	83.7
Income status			
	Good	46	22.7
	Middle	142	70.0
	Bad	15	7.4
Place of residence			
	Province	153	75.4
	District/town/village	50	24.6
Marital status			
	Married	92	45.3
	Single	111	54.7
Sexual partner			
	There is	108	53.2
	No	95	46.8
Pregnancy experience			
	No	169	83.3
	There is	34	16.7
Birth experience			
	No	181	89.2
	There is	22	10.8
Living child			
	No	181	89.2
	There is	22	10.8
Abortion experience			
	No	194	95.6
	There is	9	4.4
Low experience			
	No	186	91.6
	There is	17	8.4
Total		203	100.0

PCOS: polycystic ovary syndrome.

**Table 2:** Women's characteristics and Polycystic Ovary Syndrome Quality of Life-50 and Female Self-Confidence Scale scores.

Features		n (%)	PCOSQ-50 Scale		Female Self-Confidence Scale	
			$\bar{x}$ ± SD	Test	$\bar{x}$ ± SD	Test
Age						
	Under 25	94 (46.3%)	131.89±34.96	*1.019* *0.363*	135.54±22.97	*0.395* *0.674*
	25–30 years	75 (36.9%)	139.91±40.08		138.53±27.90	
	Over 31 years	34 (16.7%)	138.29±39.95		138.68±18.36	
PCOS diagnosis period						
	1–4 years	93 (45.8%)	140.60±39.17	*2.574* *0.079*	138.38±23.92	*1.717* *0.182*
	5–9 years	48 (23.6%)	125.58±34.15		131.63±25.24	
	10 years and over	62 (30.5%)	136.92±37.32		139.66±23.51	
Education status						
	High school and below	30 (14.8%)	139.57±37.69	*0.571* *0.569*	133.40±27.39	*-0.925* *0.356*
	University/master's degree	173 (85.2%)	135.29±37.85		137.83±23.63	
Sexual partner						
	There is	108 (53.2%)	140.01±40.93	*1.649* *0.101*	138.27±25.46	*0.687* *0.493*
	No	95 (46.8%)	131.28±33.43		135.93±22.75	
Pregnancy experience						
	No	169 (83.3%)	135.49±35.73	*-0.370* *0.712*	137.24±24.07	*0.084* *0.933*
	There is	34 (16.7%)	138.12±47.16		136.85±25.21	
Birth experience						
	No	181 (89.2%)	134.05±36.20	*-1.669* *0.108*	136.42±24.48	*-1.273* *0.205*
	There is	22 (10.8%)	151.36±47.01		143.36±21.23	
Living child						
	No	181 (89.2%)	134.05±36.20	*-1.669* *0.108*	136.42±24.48	*-1.273* *0.205*
	There is	22 (10.8%)	151.36±47.01		143.36±21.23	

PCOSQ-50: Polycystic Ovary Syndrome Quality of Life-50; SD: standard deviation; PCOS: polycystic ovary syndrome. The statistical values are represented in italics.

It was determined that there was a moderate and significant relationship between the PCOS Scale score variable and the Female Self-Confidence Scale score (R: 0.512, R^2^: 0.262, p: 0.000) and the PCOS Scale score explained 26.2% of the total variance (Table 3). In other words, the 26.2% change in the Female Self-Confidence Scale score is explained by the PCOS Scale score included in the model. According to the standardized regression coefficient, a one-unit increase in the PCOS Scale score causes a 51.2% increase in the Female Self-Confidence Scale score. The PCOS Scale score has a significant and positive effect on Female Self-Confidence Scale score.

**Table 3:** Regression analysis of polycystic ovary syndrome and Female Self-Confidence Scale.

Variables		B	Sth. error	B	T	p
(Fixed)		92.578	5.475		16.908	0.000
Polycystic Ovary Syndrome Scale score		0.328	0.039	0.512	8.452	0.000
Dependent variable: Female Self-Confidence Scale score						
	R: 0.512	R^2^: 0.262	F: 71.432	p: 0.000	Durbin-Watson: 2.024	

## DISCUSSION

This study evaluated the relationship between PCOS-related quality of life, women's self-confidence and happiness. It was found that sociodemographic and gynecologic characteristics did not significantly affect either PCOS quality of life or self-confidence levels. However, a significant correlation was observed between lower PCOS-related quality of life and decreased self-confidence.

Polycystic ovary syndrome is a multifactorial endocrine disorder that impacts not only physical health but also psychological well-being. Recent studies have shown that women with PCOS experience lower self-esteem, impaired body image^10^. For example, Soni and Laddad reported that women with PCOS exhibit increased levels of anxiety and depression, as well as greater dissatisfaction with body image compared to controls^11^. Similarly, a large Middle Eastern survey indicated that 73.9% of women with PCOS perceived themselves as unattractive, and 22.3% avoided social interactions due to appearance-related concerns^12^. Moreover, recent discussions emphasize that new insights into the definition of PCOS may help to better understand its complex psychosocial consequences^13^.

Hormonal imbalances, insulin resistance, and inflammation contribute to mood disorders such as depression and anxiety among women with PCOS^14^. These findings indicate that the syndrome affects more than physical appearance, influencing women's mental health and social interactions. Therefore, psychological care should be integrated into the treatment of PCOS.

Although the psychological impact of PCOS is well-documented, studies focusing specifically on the relationship between PCOS and self-confidence are limited. Our study aims to contribute to this gap by exploring how physical and reproductive symptoms of PCOS influence women's self-perception. In support, Jannink et al. found that infertile women with PCOS reported more anxiety and lower body image perception than infertile women without PCOS^15^. A meta-analysis by Davitadze et al. also confirmed increased body dissatisfaction in women with PCOS, highlighting the importance of psychological care in clinical practice^16^. Furthermore, a recent systematic review and meta-analysis demonstrated that PCOS has a profound effect on sexual function, underscoring the importance of addressing both physical and psychological aspects of the disorder^17^.

Li et al. observed depressive symptoms in adolescents with PCOS, emphasizing the mental health burden in younger populations^14^. In another study, Huangfu et al. reported that self-esteem and self-compassion play mediating roles between body dissatisfaction and depression, advocating for psychological interventions targeting these factors^18^. Physical changes such as obesity and hirsutism, as well as infertility-related distress, were also shown to contribute to emotional difficulties in PCOS patients^19^.

Our findings align with the existing literature, suggesting that as PCOS-related quality of life declines, self-confidence also diminishes. Wang et al. explored women's lived experiences with PCOS and identified issues including menstrual irregularities, infertility, and body dissatisfaction, all of which negatively impact mental well-being^20^. Chaudhari et al. also found that psychological disorders such as anxiety and depression are common among women with PCOS and significantly reduce quality of life^21^. Simon et al. emphasized that self-esteem issues and anxiety in PCOS patients are closely associated with poor mental health outcomes^22^.

The physical manifestations of PCOS—acne, weight gain, and hirsutism—challenge societal beauty standards and often lead to feelings of shame and inadequacy. Fertility concerns may further erode self-worth. For this reason, addressing psychological distress and promoting positive body image and self-confidence should be considered integral to PCOS management strategies.

## CONCLUSION AND RECOMMENDATIONS

Self-confidence was found to decrease as quality of life decreased in women with PCOS. Sociodemographic and gynecologic characteristics did not affect this situation. No study investigating PCOS and self-confidence was found in the literature. It is recommended that new studies should be conducted to examine the relationship between self-confidence and PCOS, which affects women in many ways, and experimental studies should be planned to increase the quality of life with PCOS to increase self-confidence.

## Data Availability

The datasets generated and/or analyzed during the current study are available from the corresponding author upon reasonable request.
